# Exploring the Causal Effects of Mineral Metabolism Disorders on Telomere and Mitochondrial DNA: A Bidirectional Two-Sample Mendelian Randomization Analysis

**DOI:** 10.3390/nu16101417

**Published:** 2024-05-08

**Authors:** Zhijun Feng, Yinghui Wang, Zhengzheng Fu, Jing Liao, Hui Liu, Meijuan Zhou

**Affiliations:** Department of Radiation Medicine, Guangdong Provincial Key Laboratory of Tropical Disease Research, School of Public Health, Southern Medical University, Guangzhou 510515, China or fengzhj18@sina.com (Z.F.); yinghui0618@163.com (Y.W.); smufuzz@163.com (Z.F.); liaojing_smu@outlook.com (J.L.); lhuidoc@163.com (H.L.)

**Keywords:** mineral metabolism disorders, iron metabolism, aging, telomere length, mitochondrial DNA copy number, Mendelian randomization

## Abstract

The aim of this study was to assess the causal relationships between mineral metabolism disorders, representative of trace elements, and key aging biomarkers: telomere length (TL) and mitochondrial DNA copy number (mtDNA-CN). Utilizing bidirectional Mendelian randomization (MR) analysis in combination with the two-stage least squares (2SLS) method, we explored the causal relationships between mineral metabolism disorders and these aging indicators. Sensitivity analysis can be used to determine the reliability and robustness of the research results. The results confirmed that a positive causal relationship was observed between mineral metabolism disorders and TL (*p* < 0.05), while the causal relationship with mtDNA-CN was not significant (*p* > 0.05). Focusing on subgroup analyses of specific minerals, our findings indicated a distinct positive causal relationship between iron metabolism disorders and both TL and mtDNA-CN (*p* < 0.05). In contrast, disorders in magnesium and phosphorus metabolism did not exhibit significant causal effects on either aging biomarker (*p* > 0.05). Moreover, reverse MR analysis did not reveal any significant causal effects of TL and mtDNA-CN on mineral metabolism disorders (*p* > 0.05). The combination of 2SLS with MR analysis further reinforced the positive causal relationship between iron levels and both TL and mtDNA-CN (*p* < 0.05). Notably, the sensitivity analysis did not indicate significant pleiotropy or heterogeneity within these causal relationships (*p* > 0.05). These findings highlight the pivotal role of iron metabolism in cellular aging, particularly in regulating TL and sustaining mtDNA-CN, offering new insights into how mineral metabolism disorders influence aging biomarkers. Our research underscores the importance of trace element balance, especially regarding iron intake, in combating the aging process. This provides a potential strategy for slowing aging through the adjustment of trace element intake, laying the groundwork for future research into the relationship between trace elements and healthy aging.

## 1. Introduction

Cellular aging is a complex biological process that profoundly impacts the health and longevity of humans [1]. In the aging process, telomere length (TL) alterations have emerged as one of the key biological markers [2,3]. A telomere is a repeating nucleotide sequence that caps the chromosomes and determines the frequency of cell division and the speed of aging [4]. Telomeres gradually shorten with cell division, creating a threshold that signals cellular aging or death [5,6,7]. Mineral and trace element metabolism also plays an important role in maintaining cellular and overall health [8,9]. Iron, in particular, is a critical mineral integral to oxygen transport, energy metabolism, and DNA synthesis [10,11]. Disruptions in iron metabolism, such as deficiency or overload, have been linked to various pathologies in chronic diseases and the aging process. Additionally, an alteration in the mitochondrial DNA copy number (mtDNA-CN) has been identified as an important marker of aging and age-related diseases in recent studies [12,13,14]. Mitochondria, as the primary source of cellular energy production [15], undergo a decline in function that is integral to the aging process [16,17,18]. The replication of mtDNA is closely tied to cellular metabolism [19]. Iron, acting as a cofactor for numerous enzymes in the respiratory chain, plays a direct role in modulating mitochondrial function [20,21]. Despite significant research efforts exploring the regulatory roles of trace elements such as iron, calcium, phosphorus, and magnesium in cellular aging and mitochondrial biology, the precise causal relationships among these elements remain incompletely understood.

This study utilizes the Mendelian randomization (MR) method to examine the causal effects of mineral metabolism disorders—specifically involving iron, calcium, phosphorus, zinc, copper, selenium, and magnesium—on TL and mtDNA-CN [22]. Our findings provide novel insights into the molecular mechanisms by which mineral metabolism influences cellular energy metabolism and aging. It provides a theoretical foundation for further exploration of the mechanisms by which mineral metabolism disorders, TL alterations, and mtDNA-CN variations contribute to aging and associated diseases.

## 2. Material and Methods

### 2.1. Study Design and Data Source

**Figure 1:**
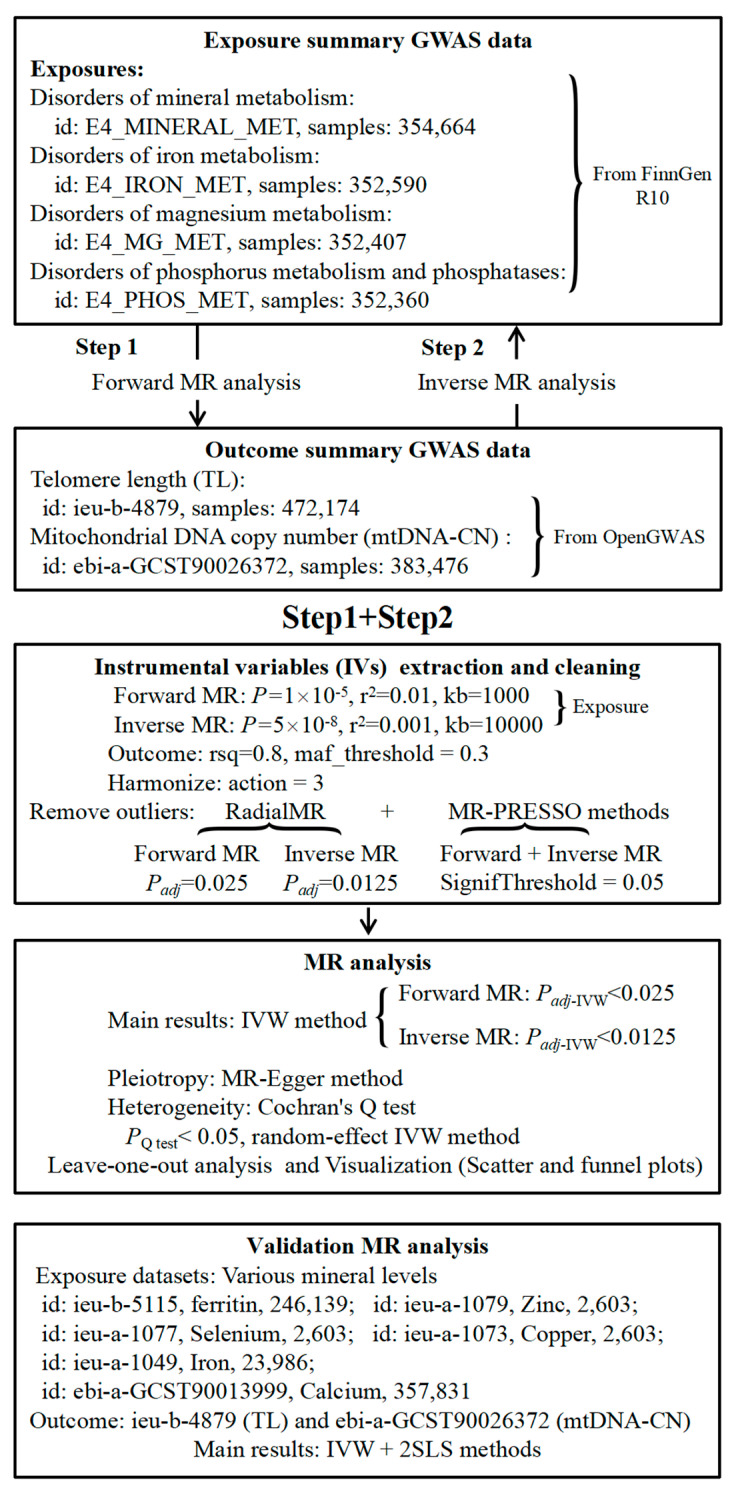
Schematic workflow of the study design and the data analysis steps. id represents the identification of genome-wide association study (GWAS) datasets; samples represents the sample size of the dataset; MR, Mendelian randomization; *P*_adj_ represents *p*-values with Bonferroni correction; IVW, inverse-variance weighted.

This is a bidirectional two-sample MR study following the STROBE-MR Statement (Strengthening the Reporting of Observational Studies in Epidemiology Using Mendelian Randomization) guidelines [23]. In Step 1, for the forward MR analysis, we identified 4 datasets associated with mineral metabolism disorders from the FinnGen database to serve as exposures [24]. These included disorders of mineral metabolism, disorders of iron metabolism, disorders of magnesium metabolism, and disorders of phosphorus metabolism and phosphatases. Detailed information about these data is provided in Figure 1. As outcomes, we selected two datasets (one is for TL [25], and the other is for mtDNA-CN [26]) from the openGWAS database (https://gwas.mrcieu.ac.uk/, accessed on 1 April 2024). In Step 2, for the reverse MR analysis, the roles of exposures and outcomes were interchanged. Based on MR analysis guidelines, rigorous criteria for procedure selection and quality control were established (Figure 1). Additionally, Bonferroni correction (BF) was applied to the corresponding *p*-values (ratio of 0.05 to the number of effective comparisons in the current analysis) [27]. Furthermore, due to the absence of mineral element level data in the FinnGen database, we utilized data from the OpenGWAS database to validate the causal effects of mineral levels on TL and mtDNA-CN, with detailed data information also provided in Figure 1.

### 2.2. Obtaining Instrumental Variables (IVs) and Data Cleaning

The single-nucleotide polymorphisms (SNPs) closely associated with exposure were selected for MR analysis according to 3 core assumptions [28]. First, IVs must exhibit a strong correlation (*p* < 1 × 10^−5^) with the exposure. Second, IVs should not be associated with any confounding factors that influence both the exposure and the outcomes. Third, IVs’ effects on the outcomes should exclusively be mediated through the exposure, without involving any indirect pathways. Based on Figure 1, we obtained IVs associated with exposure using the R package ‘TwoSampleMR’ with adequate sample size and effect allele frequency (EAF) [29,30]. The missing EAFs were supplemented using 1000 Genomes Project data [31,32]. Afterwards, *F* values for each IV were calculated (*F* = *beta*^2^/*se*^2^), and a threshold *F* value of 10 was set to include them in the analysis [33,34,35]. Furthermore, the data-cleaning procedure included the following steps: (1) Remove confounding IVs: IVs related to TL are considered confounding factors, which were identified with the ‘LDtrait’ database (https://ldlink.nih.gov/?tab=ldtrait, accessed on 1 April 2024) [36]. (2) Data harmonization: the ‘TwoSampleMR’ package was used to obtain outcome-related IVs, with parameters like proxies = T, rsq = 0.8, and maf_threshold = 0.3 [37]. These IVs were then harmonized with data of exposure. (3) Remove outliers: a combination of the ‘RadialMR’ [38] and MR-PRESSO methods [39] was used to identify and exclude outliers in this MR study. An IV with a *p*-value less than the adjusted *p*-value calculated by the ‘RadialMR’ R package or an IV with a *p* < 0.05 calculated by the ‘MR-PRESSO’ R package is considered an outlier.

### 2.3. Ethics Statement

The current research employed publicly available GWAS summary statistics data obtained from the OpenGWAS database, which obtained informed consent from all participating studies in accordance with approved protocols by their respective institutional review boards. Therefore, the inclusion of a separate ethics statement is deemed unnecessary.

### 2.4. MR Analysis

MR analysis includes both forward (various mineral metabolism to TL and mtDNA-CN) and inverse (TL and mtDNA-CN to various mineral metabolism) MR analyses. MR analysis was conducted using the ’TwoSampleMR’ R package [30] and explored the causal effects of 4 exposures on TL and mtDNA-CN. The MR analysis combined 5 methodologies [40]: MR-Egger, weighted median, inverse-variance weighting (IVW), simple mode, and weighted mode. Causality determination was based on the following rules: ① The causal effect estimate values (*B* values, also known as β values) from the 5 MR methods must exhibit directional consistency, being either all greater than 0 (positive) or all less than 0 (inverse). ② The statistical significance of the causal relationship is predominantly determined by whether the *P*_IVW_ < 0.025 (*P_adj_* = 0.05/2 (each dataset was analyzed twice)) in forward MR analysis and *P*_IVW_ < 0.00125 (*P_adj_* = 0.05/4 (each dataset was analyzed 4 times)) in inverse MR analysis [41].

### 2.5. Sensitivity Analysis

Sensitivity analysis involves analyzing heterogeneity and testing for pleiotropic effects using Cochran’s Q test and the MR–Egger method, respectively [42,43]. The MR–Egger intercept test was used to evaluate horizontal pleiotropy with a significance level of *p* < 0.05 [43]. For IVW, the fixed-effects model was used when there was no heterogeneity, and the random-effects model was used when there was heterogeneity (*P*_Q test_ < 0.05) [44]. The leave-one-out method was used to assess whether each SNP affected the effect of estimation. Funnel plots and scatter plots of MR analysis were used to visually assess the levels of pleiotropy and heterogeneity.

### 2.6. Validation MR Analysis

The impact of different mineral levels on TL and mtDNA-CN was re-evaluated utilizing aggregated genome-wide association study (GWAS) data. The exposure data were sourced from the openGWAS database, comprising GWAS information on a range of mineral elements including selenium [45], copper [45], iron [46], and calcium [47], as well as ferritin levels (primarily a storage form of iron) [48]. Details of datasets are listed in Figure 1. Noteworthy is that the zinc, copper, and selenium data were obtained from an Australian cohort, while the remaining data were sourced from summary GWAS datasets of European descent. It is crucial to note that the validation analysis primarily utilized the IVW method in conducting MR, supplemented by the two-stage least squares (2SLS) method [28,49]. The presence of a substantial causal relationship was ascertained by both methods producing *p*-values less than 0.05. Furthermore, sensitivity analyses and corresponding visualization processes were also performed.

## 3. Results

### 3.1. The Causal Effect Estimates of Mineral Metabolism Disorders on TL and mtDNA-CN

**Figure 2:**
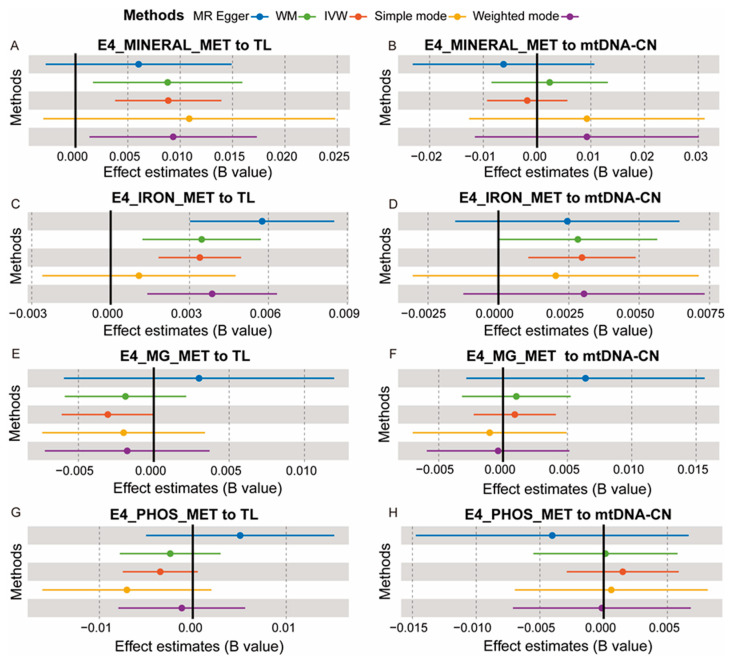
The causal effect estimates of mineral metabolism disorders on telomere length (TL) and mitochondrial DNA copy number (mtDNA-CN). E4_MINERAL_MET, disorders of mineral metabolism; E4_IRON_MET, disorders of iron metabolism; E4_MG_MET, disorders of magnesium metabolism; E4_PHOS_MET, disorders of phosphorus metabolism and phosphatases. The dots represent the overall causal estimation in the analysis direction, the horizontal lines represent the upper (**right**) and lower (**left**) limits of effect estimation, and different-colored lines represent different MR analysis methods. WM, weight median; IVW, inverse-variance weighted.

SNPs associated with the four mineral metabolism disorders were extracted as IVs, and Tables S1–S4 (Table S1 for disorders of mineral metabolism, Table S2 for disorders of iron metabolism, Table S3 for disorders of magnesium metabolism, Table S4 for disorders of phosphorus metabolism and phosphatase metabolism) in the Supplementary Materials provides detailed information about these IVs. In each analysis direction, the IV had an *F* value greater than 10. Based on the results of forward MR analysis, a positive causal relationship was revealed between disorders of mineral metabolism and TL (*B* = 0.009, *P*_IVW_ = 6.19 × 10^−4^, Figure 2A). However, no significant causal effects were observed between mineral metabolism disorders and mtDNA-CN (*P*_IVW_ = 0.63, Figure 2B). The result also indicates a positive causal impact of iron metabolism disorders on both TL (*B* = 0.003, *P*_IVW_ = 2.29 × 10^−5^, Figure 2C) and mtDNA-CN (*B* = 0.003, *P*_IVW_ = 2.28E × 10^−3^, Figure 2D). There were no significant causal associations found for disorders of magnesium metabolism either on TL (*P*_IVW_ = 0.05, Figure 2E) or on mtDNA-CN (*P*_IVW_ = 0.58, Figure 2F). Additionally, disorders involving phosphorus metabolism and phosphatases showed no significant effects on TL (*P*_IVW_ = 0.09, Figure 2G) or mtDNA-CN (*P*_IVW_ = 0.50, Figure 2H). A detailed description of the forward MR analysis results can be found in Table S5. This evidence reveals a mechanistic link between mineral metabolism and the biological process of cellular aging, particularly in cases of iron metabolism disorders. Conversely, the lack of substantial causal relationships in instances of magnesium and phosphorus metabolism disorders underscores the specificity of mineral-related impacts on cellular aging markers.

### 3.2. The Causal Effect Estimates of Mineral Metabolism Disorders on TL and mtDNA-CN

SNPs associated with TL and mtDNA-CN were identified as IVs. Detailed information on these IVs can be found in Tables S6 and S7 of the Supplementary Materials, with Table S6 related to TL and Table S7 to mtDNA-CN. Each IV exhibited an *F* value exceeding 10 in every analysis direction. Despite performing inverse MR analysis, no significant causal effect was observed in any analysis direction, as indicated by the *p*-value associated with the IVW method exceeding 0.05 in all analysis directions (Table 1). Further details on the inverse MR results are provided in Table S8. This evidence indicates that there is an independent relationship between mineral metabolism disorders and body aging, indicating that they are not causal outcomes of aging.

### 3.3. Sensitivity Analysis

**Figure 3:**
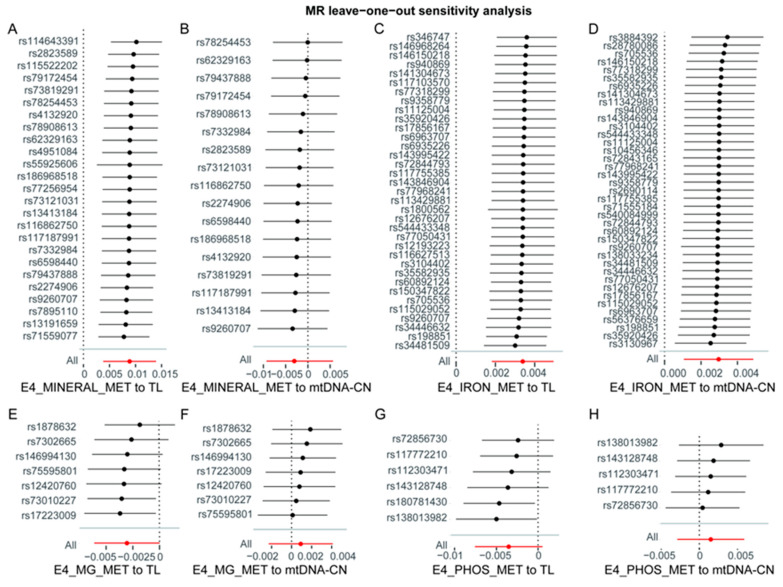
Leave-one-out analysis to assess the effect of each SNP in driving causality. E4_MINERAL_MET, disorders of mineral metabolism; E4_IRON_MET, disorders of iron metabolism; E4_MG_MET, disorders of magnesium metabolism; E4_PHOS_MET, disorders of phosphorus metabolism and phosphatases. TL, telomere length; mtDNA-CN, mitochondrial DNA copy number.

**Figure 4:**
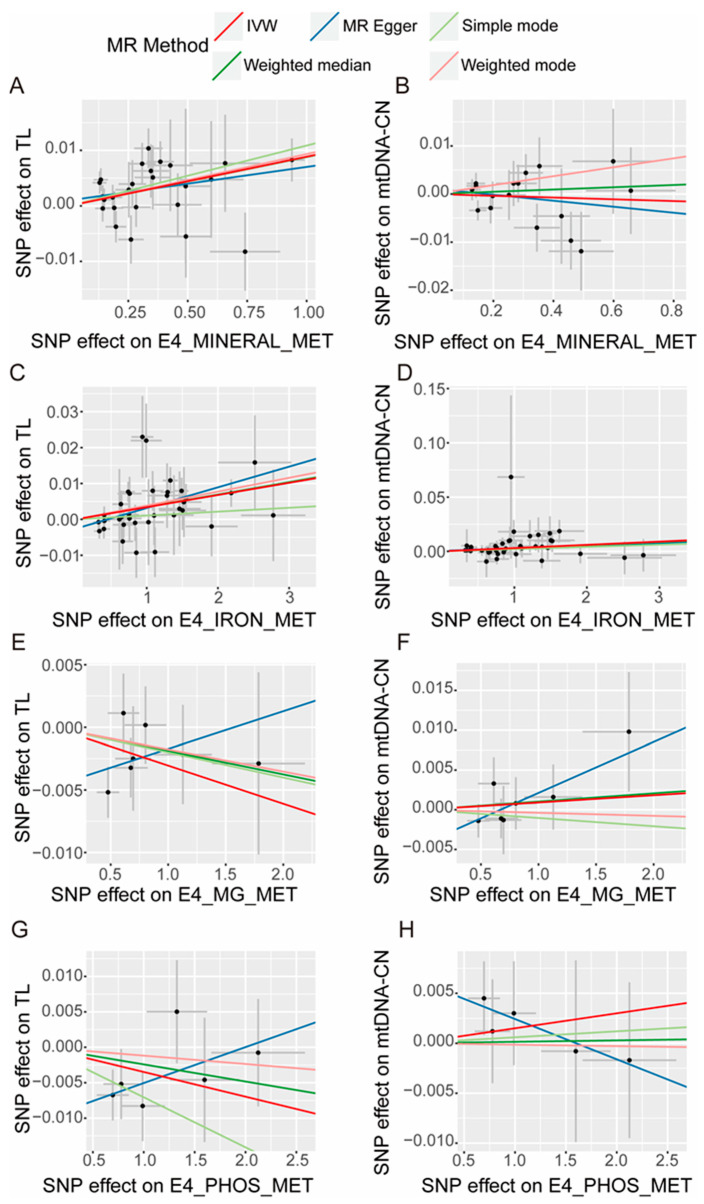
Scatter plots of SNP effects on the telomere length (TL), mitochondrial DNA copy number (mtDNA-CN), and the various mineral metabolism disorders. E4_MINERAL_MET, disorders of mineral metabolism; E4_IRON_MET, disorders of iron metabolism; E4_MG_MET, disorders of magnesium metabolism; E4_PHOS_MET, disorders of phosphorus metabolism and phosphatases. The dots represent SNP for causal estimation in the analysis direction; the different-colored lines represent different MR analysis methods. IVW, inverse-variance weighted.

**Figure 5:**
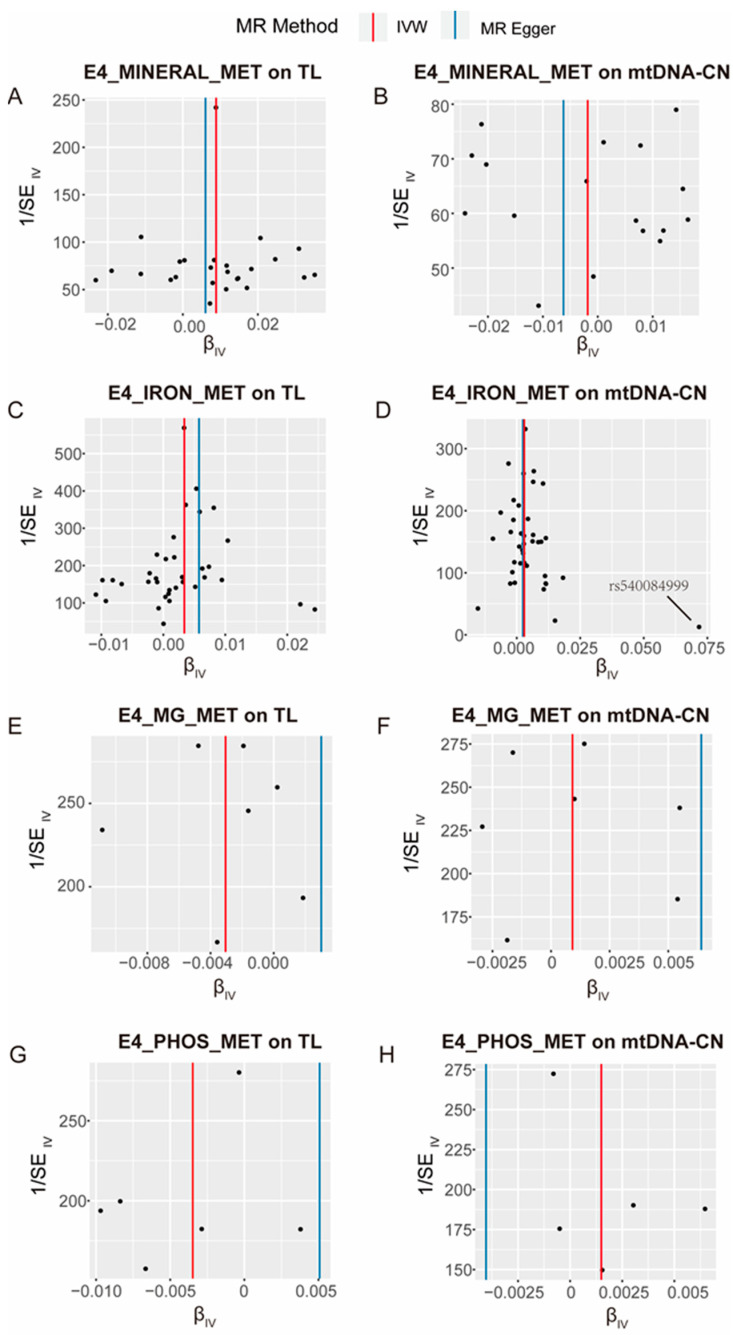
Funnel plots of the effect size against the inverse of the standard error (SE) for each SNP and the various mineral metabolism disorders. E4_MINERAL_MET, disorders of mineral metabolism; E4_IRON_MET, disorders of iron metabolism; E4_MG_MET, disorders of magnesium metabolism; E4_PHOS_MET, disorders of phosphorus metabolism and phosphatases. The dots represent SNPs for causal estimation in the analysis direction; the different-colored lines represent different MR analysis methods. TL, telomere length; mtDNA-CN, mitochondrial DNA copy number; IVW, inverse-variance weighted.

The sensitivity analysis involved assessing heterogeneity through Cochran’s Q test and pleiotropy through the MR–Egger test. The findings suggested that there is no statistically significant evidence of either heterogeneity (Table S9) or pleiotropy (Table S10) in the forward and inverse MR analyses. The leave-one-out test found a trend of causal effect changes after individually removing SNPs in each analysis direction, both forward and inverse analyses. No significantly abnormal SNPs were found in these directions (Figure 3 for forward analysis and Figures S1–S8 for inverse analysis). For forward analysis, scatter plots (Figure 4) visually represent the causal relationships between mineral metabolism disorders and TL, as well as mtDNA-CN. The effect of SNPs on exposures and outcomes shows a clear linear trend in those directions where MR analysis has statistical significance (Figure 4A for mineral metabolism disorders on TL, Figure 4C for iron metabolism disorders on TL, and Figure 4D for iron metabolism disorders on mtDNA-CN). Funnel plots (Figure 5) show symmetrical effect size variations around estimated causal effects, except for the direction of iron metabolism disorders on mtDNA-CN. Although the funnel plot in this direction shows a significant abnormal distribution of rs540084999, according to the result of the leave-one-out test, the causal effect estimation was not changed after removing rs540084999. Therefore, rs540084999 is not a potential outlier. For the inverse MR analysis, the scatter plots (Figure S9) and funnel plots (Figure S10) are provided in the Supplementary Materials, and no SNPs with abnormal distributions were found. According to these results, IVs included in the current analysis do not have directional pleiotropic effects or heterogeneity, and the results of this study are robust.

### 3.4. Validation Analysis of the Causal Effect of Different Mineral Levels on TL and mtDNA-CN

**Figure 6:**
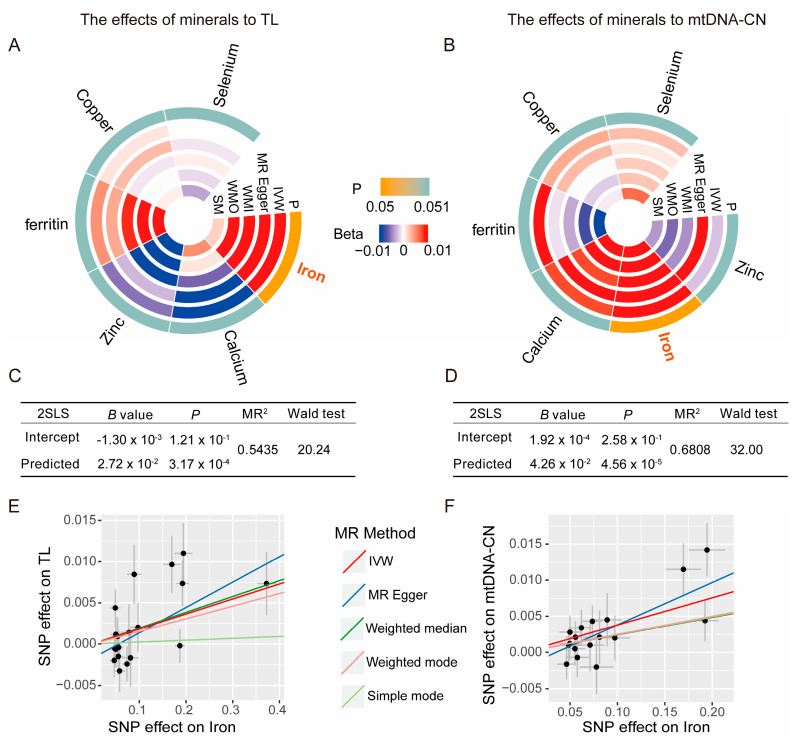
Mendelian randomization (MR) combined 2-stage least squares (2SLS) analysis of the causal effects of mineral content levels on telomere length (TL) and mitochondrial DNA copy number (mtDNA-CN). (**A**,**B**) Results of MR analysis; (**C**,**D**) results of 2SLS method; (**E**,**F**) scatter plots of SNP effects. MR^2^, Multiple R-Squared; IVW, inverse-variance weighted; WMI, weighted median; WMO, weighted mode; SM, simple mode.

Validation analysis was conducted to explore the causal effects of different mineral levels on TL and mtDNA-CN. We applied the same quality control criteria and procedure as forward MR analysis. In combination with the 2SLS method, an MR analysis was used to verify the causal relationship. The MR analysis indicated positive causal relationships between iron levels and TL (Figure 6A, *B* = 0.018, *P*_IVW_ = 7.22 × 10^−3^), as well as iron levels and mtDNA-CN (Figure 6B, *B* = 0.008, *P*_IVW_ = 5.78 × 10^−7^). Similar results were obtained with the 2SLS method (Figure 6C, iron levels to TL, *B* = 0.027, *p* = 3.17 × 10^−4^; Figure 6D, iron levels to mtDNA-CN, *B* = 0.043, *p* = 4.56 × 10^−5^). The explanatory power of iron levels on TL (MR^2^ = 0.5435) and mtDNA-CN exceeds 50%, especially with an explanatory power of nearly 70% on mtDNA-CN (MR^2^ = 0.6808), which further highlights the importance of iron’s influence on TL and mtDNA-CN. The sensitivity analysis in these directions did not reveal any significant statistics (Table S12, iron to TL, *P*_Q test_ = 0.06, *P*_egger_intercept_ = 0.17; iron to mtDNA-CN, *P*_Q test_ = 0.71, *P*_egger_intercept_ = 0.14), and scatter plots (Figure 6E for iron levels to TL, Figure 6F for iron levels to mtDNA-CN) revealed any unusual distributions of values. This evidence suggests that iron positively influences TL and mtDNA-CN, thus highlighting its importance in the aging process.

## 4. Discussion

In this study, bidirectional two-sample MR analysis was employed to assess the potential causal relationship between disorders of mineral metabolism and TL and mtDNA-CN. The results of the MR analysis indicate a positive causal relationship between disorders of mineral metabolism and TL, but no significant relationship between disorders of mineral metabolism and mtDNA-CN. In light of the fact that TL is an indicator of cellular aging [50,51], these findings suggest that an increase in disorders of mineral metabolism may contribute to the maintenance of TL, thereby influencing the aging process and potentially increasing vulnerability to diseases associated with TL prolongation. This finding underscores the significance of mineral metabolism in preserving optimal TLs and retarding the aging process. Furthermore, the stratified analyses demonstrated a significant positive causal association between disorders of iron metabolism and TL and mtDNA-CN, while no significant causal effects were observed for disorders of magnesium and phosphorus metabolism on TL and mtDNA-CN. Subsequent validation analysis confirmed the positive causal impact of iron levels on TL and mtDNA-CN and indicated that calcium, zinc, selenium, and copper levels did not have a significant causal influence on the relationship between TL and mtDNA-CN. The reverse MR analysis of TL and mtDNA-CN on disorders of mineral metabolism did not show any significant causal effects. These findings demonstrate strong robustness, as the sensitivity analysis of the IVs utilized in the current study did not indicate significant heterogeneity or pleiotropy. These findings underscore the significant role of mineral metabolism in cellular aging, particularly in maintaining TL and mtDNA-CN. Meanwhile, these results highlight the criticality of iron metabolism in cellular health and function, suggesting that the balance of iron metabolism may be a key factor influencing cellular aging and associated disease risks.

The focus of the results from this study is on iron metabolism. This study found significant effects of iron levels and iron metabolism disorders on outcomes; however, the causal effect of ferritin on outcomes did not prove statistically significant. This evidence suggests that while overall iron metabolism plays a crucial role in influencing the outcomes studied, the specific impact of ferritin levels may be more complex or indirect. Ferritin reflects the body’s iron reserves rather than its active metabolic state [52,53,54]. This differentiation may explain the lack of a direct statistical significance in the relationship between ferritin levels and the outcomes. Therefore, the role of iron in the process of cellular aging appears to be more intricately related to its metabolic activity and regulatory mechanisms, rather than merely its storage or concentration in the body. Iron, a vital micronutrient in the human body, serves various essential functions, particularly in oxygen transport and energy metabolism [55,56,57]. The regulation of iron homeostasis, encompassing absorption, utilization, storage, and excretion, is a tightly controlled process [58,59]. Iron plays a crucial role in the formation of hemoglobin and myoglobin, which are essential for the transportation of oxygen in the body [60,61]. This function is vital for the maintenance of tissue oxygenation and cellular energy production [62,63]. In energy metabolism, iron acts as a cofactor for a variety of enzymes [64], particularly within the mitochondria, where it plays a critical role in multiple stages of the electron transport chain, which is necessary for the production of ATP [65,66,67]. Telomeres, specialized structures located at the termini of chromosomes, play a crucial role in preserving chromosomal stability and safeguarding genetic integrity [68,69,70]. Their gradual shortening during cell division can lead to cellular aging or programmed cell death, known as apoptosis, upon reaching a critical length [68,71,72]. Iron serves as a cofactor for numerous pivotal enzymes engaged in DNA replication and repair processes [73,74,75]. Particularly noteworthy is its indispensable role in ribonucleotide reductase (RNR), where iron contributes significantly to regulating the deoxyribonucleotide (dNTP) pool essential for telomeric DNA synthesis [76,77]. Consequently, elevated iron levels may contribute to TL maintenance by increasing the activity of these enzymes. Additionally, mitochondria, serving as the primary energy generators of cells, play a pivotal role in cellular metabolism and the synthesis of energy [15,16,18]. The preservation of mtDNA-CN is intricately linked to the metabolic status of the cell. Iron is involved in numerous essential biochemical processes within the mitochondria, particularly in the electron transport chain [78,79,80]. Elevated levels of iron may augment mitochondrial function, thereby bolstering mtDNA replication and amplifying its quantity. Additionally, iron plays a crucial role in the activation of certain antioxidant enzymes, including superoxide dismutase (SOD), which aids in mitigating oxidative stress and safeguarding mtDNA integrity [81,82]. Nevertheless, the excessive accumulation of iron can result in detrimental consequences, including heightened oxidative stress that may compromise mitochondrial integrity and functionality, ultimately hastening cellular senescence [83,84,85]. Oxidative stress, characterized by the deleterious impact of reactive species such as free radicals on cellular components such as lipids, proteins, and DNA, can be exacerbated by iron due to its role as a potent reductant that catalyzes the generation of additional free radicals [86,87]. Consequently, an abundance of iron has been linked to the pathogenesis of several neurodegenerative disorders, such as Alzheimer’s [88], Parkinson’s disease [89,90], and other diseases of neuronal degeneration [91,92,93]. The dysregulation of iron levels, whether in excess or deficiency, can result in cellular functional imbalance, thereby hastening the aging process and potentially contributing to the onset of related diseases [94,95,96]. Investigating the interplay between iron and cellular aging offers valuable insights into the mechanisms underlying aging and presents opportunities for the development of novel therapeutic interventions. Subsequent research endeavors should delve into the intricacies of iron metabolism across various biological pathways and explore the potential benefits of modulating iron homeostasis in promoting overall health and extending lifespan. As a result of these efforts, we can anticipate more effective management of iron metabolic disorders as well as novel therapeutic avenues for slowing aging and improving age-related diseases.

While the evidence from our study appears robust and reliable based on the sensitivity analysis results, there are still limitations that warrant attention. Firstly, the observational nature of the study design may allow potential confounding factors to affect causal inferences, despite our efforts to control for known confounders. Secondly, the datasets used in this study might not fully represent the entire population, especially across different racial and cultural backgrounds; thus, caution is needed when generalizing the results. Additionally, our analysis primarily focused on overall iron levels, not delving into the differential impacts of various types of iron, such as free or bound iron, on the biological outcomes. Environmental factors like individual lifestyle choices and dietary habits, which can influence iron metabolism, were not considered in our study. Thirdly, the impact of sample overlap needs consideration. Although we utilized population samples from different database sources for the forward and reverse analysis, minimizing the bias from sample overlap, subsequent validation analyses were confined by the lack of mineral level data in the FinnGen database. We thus resorted to the summary GWAS data from the openGWAS database, with samples for zinc, selenium, and copper originating from an Australian cohort, helping to avoid overlap with the outcome samples. However, data for ferritin, iron levels, and calcium levels did involve partial sample overlap with the outcomes, potentially impacting the results.

## 5. Conclusions

The results of the MR analysis confirmed a positive causal effect between mineral metabolism disorders, particularly iron metabolism disorders, and both TL and mtDNA-CN. Conversely, no causal effect was observed from TL and mtDNA-CN on mineral metabolism disorders. These findings highlight the pivotal role of iron metabolism in cellular aging, particularly in regulating TL and sustaining mtDNA-CN, offering new insights into how mineral metabolism disorders influence aging biomarkers. Our research underscores the importance of trace element balance, especially regarding iron intake, in combating the aging process. This provides a potential strategy for slowing aging through the adjustment of trace element intake, laying the groundwork for future research into the relationship between trace elements and healthy aging.

## Figures and Tables

**Table 1:** The causal effect estimates of telomere length (TL) and mitochondrial DNA copy number (mtDNA-CN) on mineral metabolism disorders.

Methods	*N*snp	*B*	*SE*	*p*	*B*_low_	*B*_up_
TL to E4_MINERAL_MET						
MR–Egger	115	−0.085	0.205	0.680	−0.488	0.317
Weighted median	115	0.154	0.192	0.423	−0.223	0.532
IVW	115	0.022	0.120	0.857	−0.214	0.257
TL to E4_IRON_MET						
MR–Egger	114	−0.879	0.552	0.114	−1.960	0.202
Weighted median	114	−1.008	0.518	0.052	−2.025	0.008
IVW	114	−0.625	0.324	0.054	−1.259	0.010
TL to E4_MG_MET						
MR–Egger	116	0.285	0.759	0.708	−1.203	1.773
Weighted median	116	0.437	0.732	0.551	−0.998	1.872
IVW	116	−0.326	0.443	0.462	−1.194	0.543
TL to E4_PHOS_MET						
MR–Egger	115	−0.684	0.912	0.455	−2.472	1.104
Weighted median	115	−0.225	0.825	0.785	−1.843	1.392
IVW	115	−0.395	0.532	0.458	−1.438	0.648
mtDNA-CN to E4_MINERAL_MET						
MR Egger	53	0.005	0.476	0.992	−0.928	0.938
Weighted median	53	−0.157	0.324	0.627	−0.792	0.477
IVW	53	0.086	0.208	0.679	−0.322	0.495
mtDNA-CN to E4_IRON_MET						
MR–Egger	52	2.072	1.245	0.102	−0.368	4.512
Weighted median	52	0.240	0.852	0.778	−1.429	1.910
IVW	52	0.506	0.547	0.354	−0.565	1.578
mtDNA-CN to E4_MG_MET						
MR–Egger	53	−0.637	1.753	0.718	−4.072	2.798
Weighted median	53	0.146	1.212	0.904	−2.230	2.522
IVW	53	0.696	0.771	0.367	−0.815	2.206
mtDNA-CN to E4_PHOS_MET						
MR–Egger	53	−2.423	2.080	0.249	−6.499	1.653
Weighted median	53	−1.657	1.379	0.230	−4.360	1.046
IVW	53	−1.040	0.908	0.252	−2.819	0.739

E4_MINERAL_MET, disorders of mineral metabolism; E4_IRON_MET, disorders of iron metabolism; E4_MG_MET, disorders of magnesium metabolism; E4_PHOS_MET, disorders of phosphorus metabolism and phosphatases. *N*snp, number of single nucleotide polymorphisms (SNPs); *B*, causal effect estimate; *SE*, standard error; *B*_low_, the lower limits of effect estimation; *B*_up_, the upper limits of effect estimation; IVW, inverse-variance weighted.

## Data Availability

All data used in this study are available in the public repository. The code involved in the data analysis process can be obtained by contacting the corresponding author.

## Supplementary Materials

The following supporting information can be downloaded at: https://www.mdpi.com/article/10.3390/nu16101417/s1, Figure S1: MR leave−one−out sensitivity analysis for TL on minral met; Figure S2: MR leave-one-out sensitivity analysis for TL on iron met; Figure S3: MR leave-one-out sensitivity analysis for TL on MGmet; Figure S4: MR leave-one-out sensitivity analysis for TL on PHOS met; Figure S5: MR leave-one-out sensitivity analysis for mtDNA CN on minral met; Figure S6: MR leave-one-out sensitivity analysis for mtDNA CN on lRON met; Figure S7: MR leave-one-out sensitivity analysis for mtDNA CN on MG met; Figure S8: MR leave-one-out sensitivity analysis for mtDNA CN on PHOS met; Figure S9: scatter plot for inverse MR analysis; Figure S10: Funnel plot for inverse MR analysis; Table S1: Instrumental variables related to finngen R10 E4 MINERAL MET and added F values; Table S2: Instrumental variables related to finngen R10 E4 IRON MET and added F values; Table S3: Instrumental variables related to finngen R10 E4 MG MET and added F values; Table S4: Instrumental variables related to finngen R10 E4 PHOS MET and added F values; Table S5: All results of forward MR analyses; Table S6: Instrumental variables related to ieu-b-4879 and added F values; Table S7: Instrumental variables related to ebi-a-GCST90026372 and added F Values; Table S8: All results of inverse MR analysis; Table S9: heterogeneity_results; Table S10: Pleiotropy_results; Table S11: All MR results for validation datasets; Table S12: Heterogeneity and pleiotropy test.
