# Supplementary material for: Exploring the Causal Effects of Mineral Metabolism Disorders on Telomere and Mitochondrial DNA: A Bidirectional Two-Sample Mendelian Randomization Analysis
Source: Nutrients. 2024 May 8;16(10):1417. doi: 10.3390/nu16101417 (PMC11123946; doi:10.3390/nu16101417)
Supplement: Supplementary file 1 [file nutrients-16-01417-s001.zip › Figures/Figure S10 Funnel plot for inverse MR .pdf]

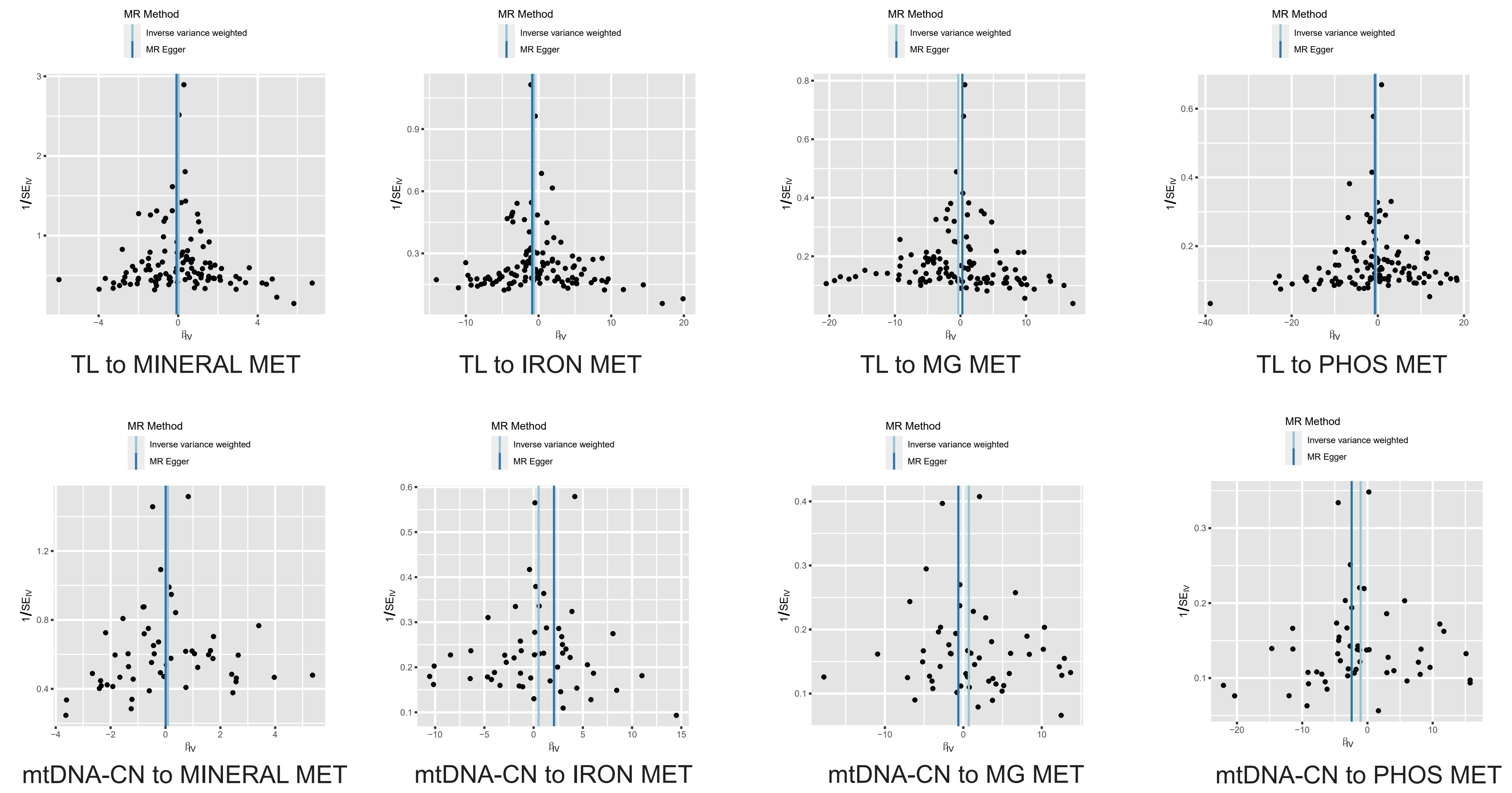
MR Method
Inverse variance weighted
MR Egger
$1/SE_{IV}$
β_{IV}
TL to MINERAL MET
TL to IRON MET
TL to MG MET
TL to PHOS MET
mtDNA-CN to MINERAL MET
mtDNA-CN to IRON MET
mtDNA-CN to MG MET
mtDNA-CN to PHOS MET
