# Supplementary material for: Exploring the Causal Effects of Mineral Metabolism Disorders on Telomere and Mitochondrial DNA: A Bidirectional Two-Sample Mendelian Randomization Analysis
Source: Nutrients. 2024 May 8;16(10):1417. doi: 10.3390/nu16101417 (PMC11123946; doi:10.3390/nu16101417)
Supplement: Supplementary file 1 [file nutrients-16-01417-s001.zip › Figures/Figure S7 mtDNA_CN to MG met.pdf]

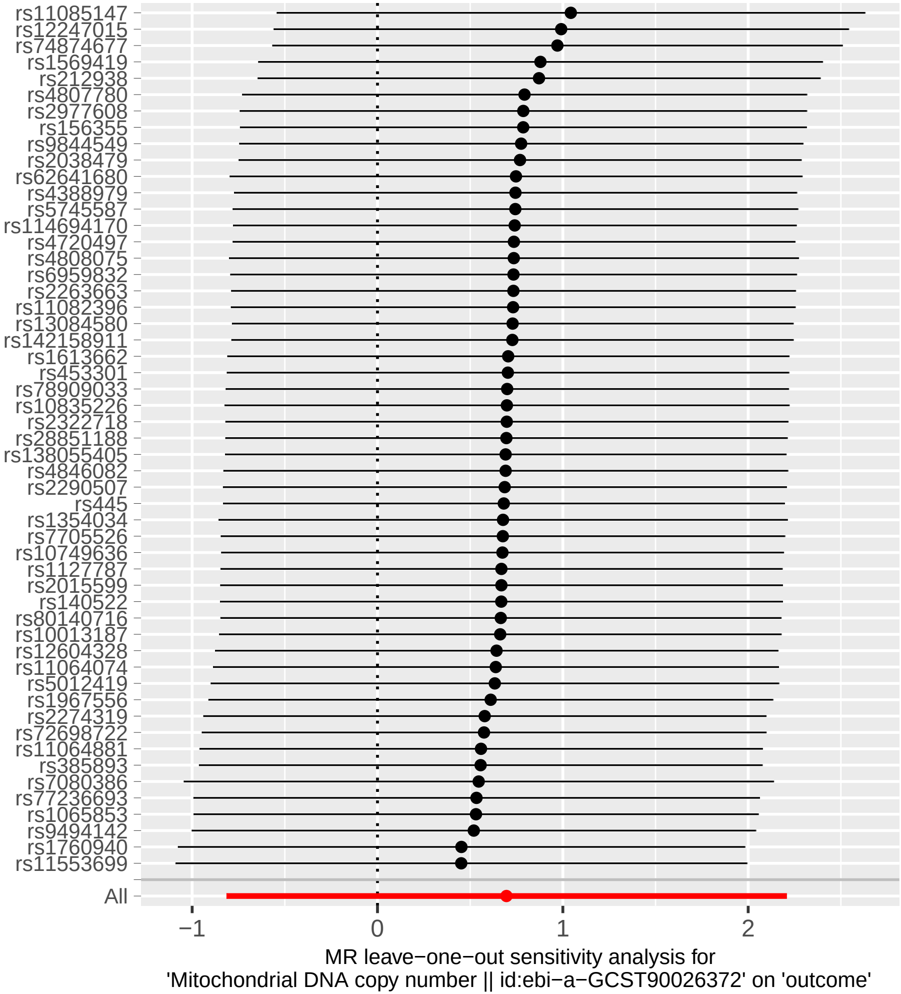
rs11085147
rs12247015
rs74874677
rs1569419
rs212938
rs4807780
rs2977608
rs156355
rs9844549
rs2038479
rs62641680
rs4388979
rs5745587
rs114694170
rs4720497
rs4808075
rs6959832
rs2263663
rs11082396
rs13084580
rs142158911
rs1613662
rs453301
rs78909033
rs10835226
rs2322718
rs28851188
rs138055405
rs4846082
rs2290507
rs445
rs1354034
rs7705526
rs10749636
rs1127787
rs2015599
rs140522
rs80140716
rs10013187
rs12604328
rs11064074
rs5012419
rs1967556
rs2274319
rs72698722
rs11064881
rs385893
rs7080386
rs77236693
rs1065853
rs9494142
rs1760940
rs11553699
All
−1
0
1
2
MR leave−one−out sensitivity analysis for
'Mitochondrial DNA copy number || id:ebi−a−GCST90026372' on 'outcome'
