# Supplementary material for: Exploring the Causal Effects of Mineral Metabolism Disorders on Telomere and Mitochondrial DNA: A Bidirectional Two-Sample Mendelian Randomization Analysis
Source: Nutrients. 2024 May 8;16(10):1417. doi: 10.3390/nu16101417 (PMC11123946; doi:10.3390/nu16101417)
Supplement: Supplementary file 1 [file nutrients-16-01417-s001.zip › Figures/Figure S8 mtDNA_CN to PHOS met.pdf]

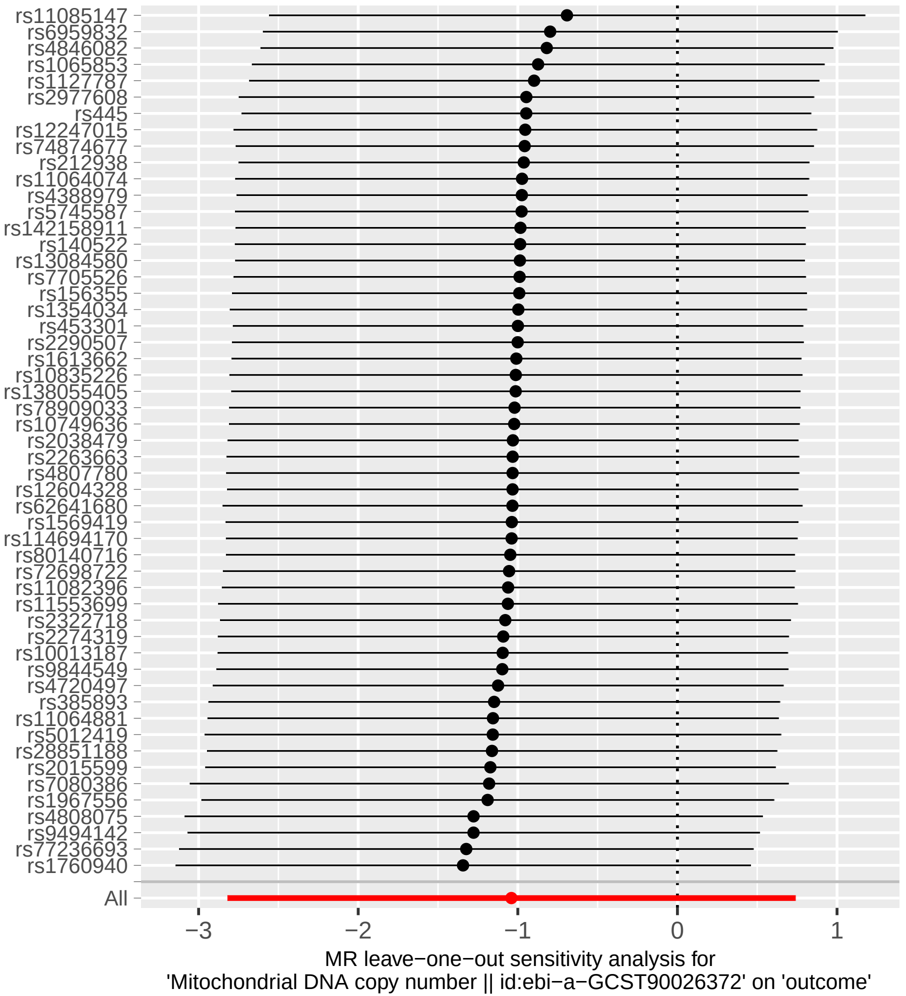
rs11085147
rs6959832
rs4846082
rs1065853
rs1127787
rs2977608
rs445
rs12247015
rs74874677
rs212938
rs11064074
rs4388979
rs5745587
rs142158911
rs140522
rs13084580
rs7705526
rs156355
rs1354034
rs453301
rs2290507
rs1613662
rs10835226
rs138055405
rs78909033
rs10749636
rs2038479
rs2263663
rs4807780
rs12604328
rs62641680
rs1569419
rs114694170
rs80140716
rs72698722
rs11082396
rs11553699
rs2322718
rs2274319
rs10013187
rs9844549
rs4720497
rs385893
rs11064881
rs5012419
rs28851188
rs2015599
rs7080386
rs1967556
rs4808075
rs9494142
rs77236693
rs1760940
All
−3
−2
−1
0
1
MR leave−one−out sensitivity analysis for
'Mitochondrial DNA copy number || id:ebi−a−GCST90026372' on 'outcome'
