# Supplementary material for: Exploring the Causal Effects of Mineral Metabolism Disorders on Telomere and Mitochondrial DNA: A Bidirectional Two-Sample Mendelian Randomization Analysis
Source: Nutrients. 2024 May 8;16(10):1417. doi: 10.3390/nu16101417 (PMC11123946; doi:10.3390/nu16101417)
Supplement: Supplementary file 1 [file nutrients-16-01417-s001.zip › Figures/Figure S9 scatter plot for inverse MR.pdf]

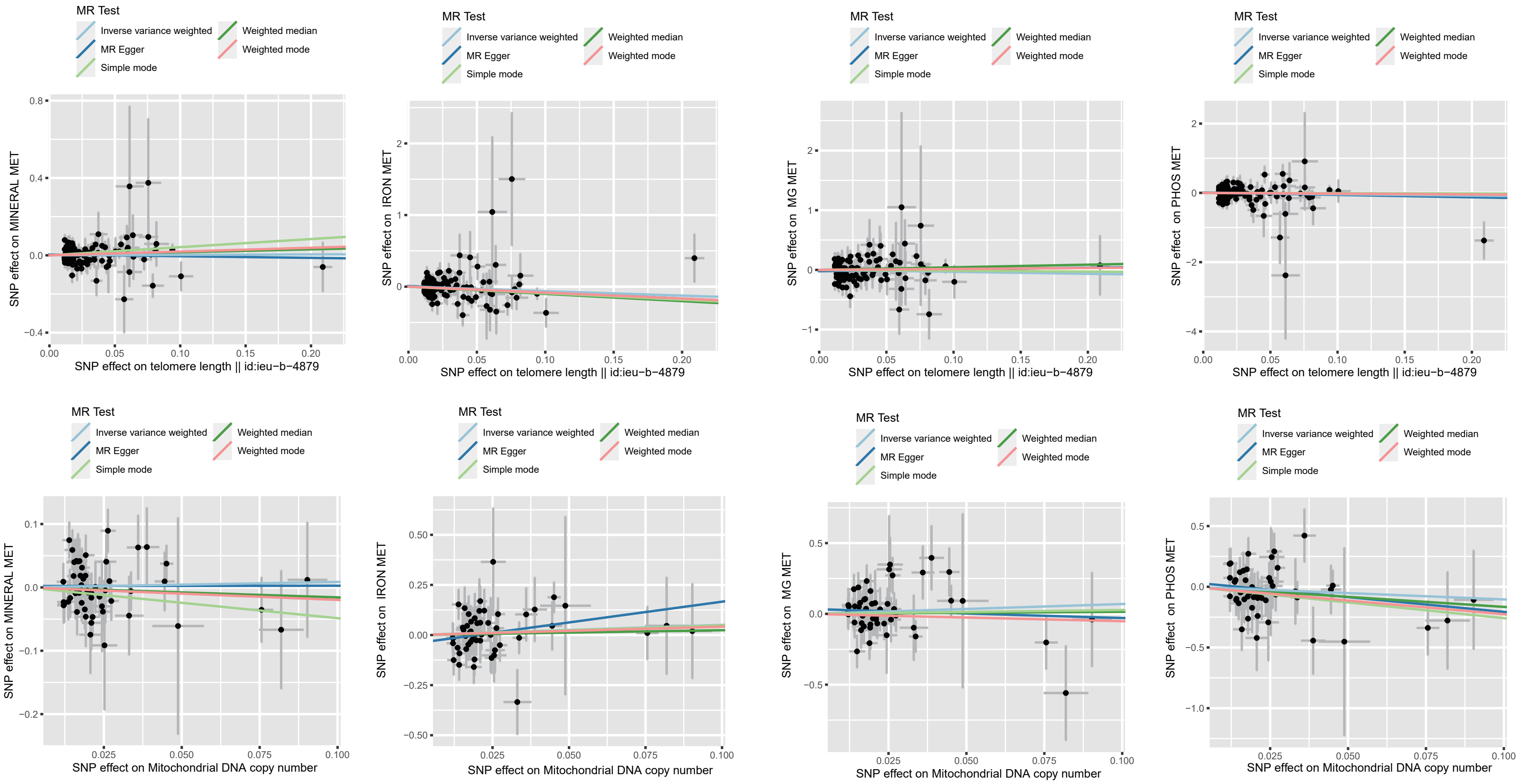
MR Test
Inverse variance weighted
MR Egger
Simple mode
Weighted median
Weighted mode
SNP effect on MINERAL MET
SNP effect on IRON MET
SNP effect on MG MET
SNP effect on PHOS MET
SNP effect on telomere length || id:ieu−b−4879
SNP effect on Mitochondrial DNA copy number
