# Supplementary figures and images for: Exploring the Causal Effects of Mineral Metabolism Disorders on Telomere and Mitochondrial DNA: A Bidirectional Two-Sample Mendelian Randomization Analysis
Source: Nutrients. 2024 May 8;16(10):1417. doi: 10.3390/nu16101417 (PMC11123946; doi:10.3390/nu16101417)

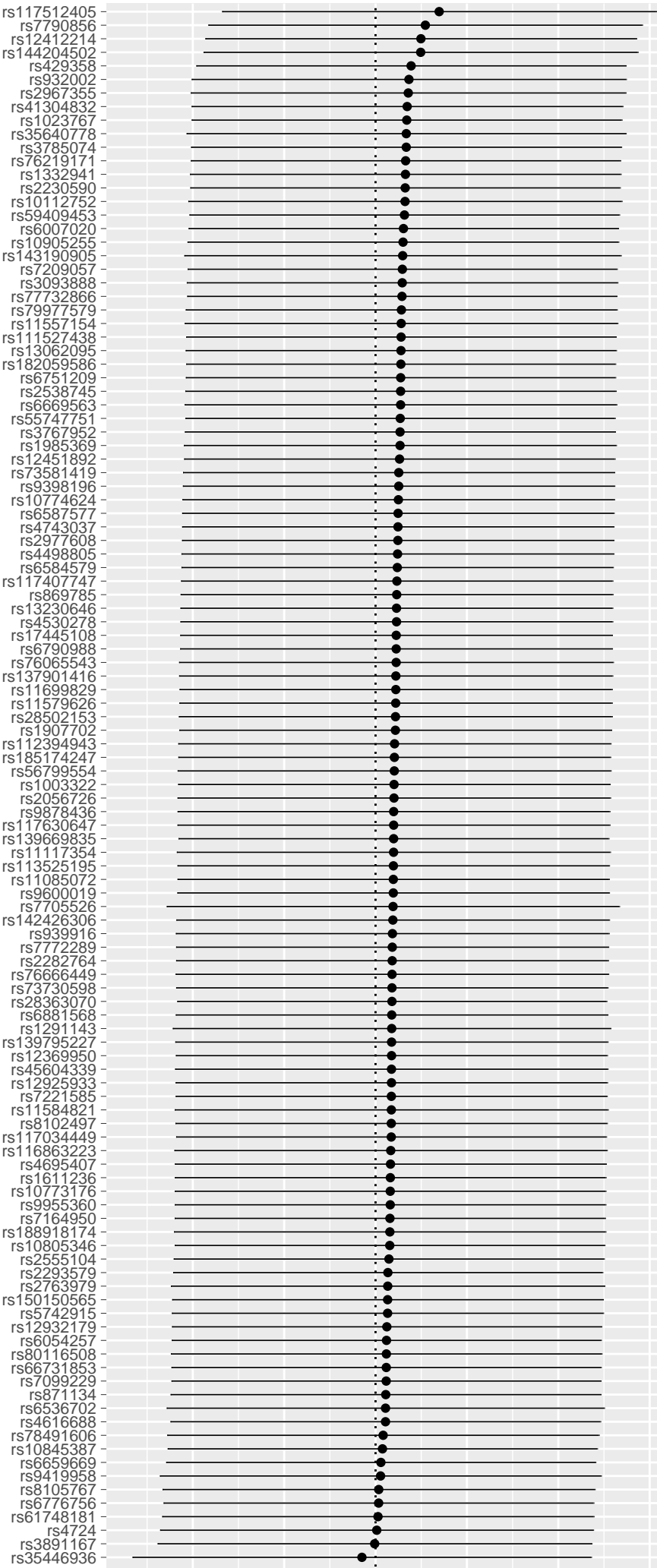

All

MR leave-one-out sensitivity analysis for 'telomere length || id:ieu-b-4879' on 'outcome'

Supplement: Supplementary file 1 [file nutrients-16-01417-s001.zip › Figures/Figure S1 TL to minral met.pdf]

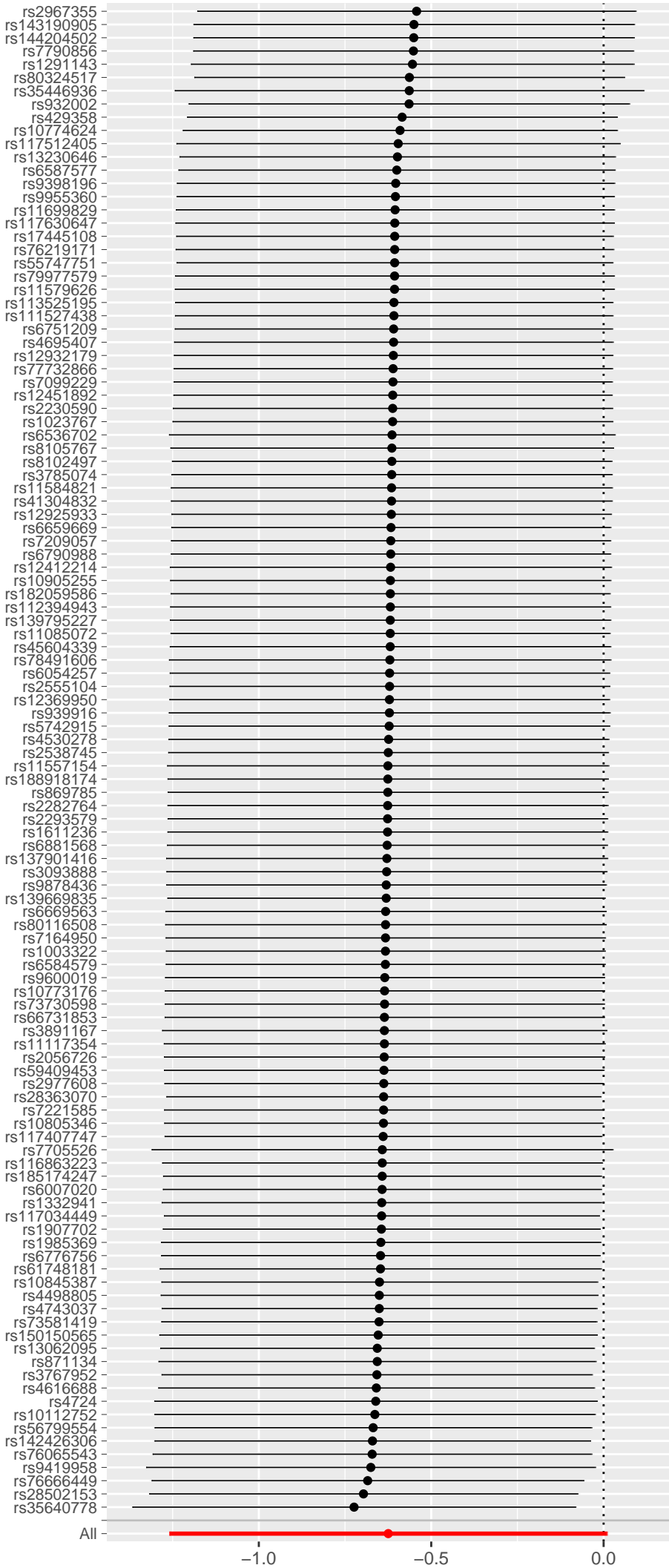

Supplement: Supplementary file 1 [file nutrients-16-01417-s001.zip › Figures/Figure S2 TL to iron met.pdf]

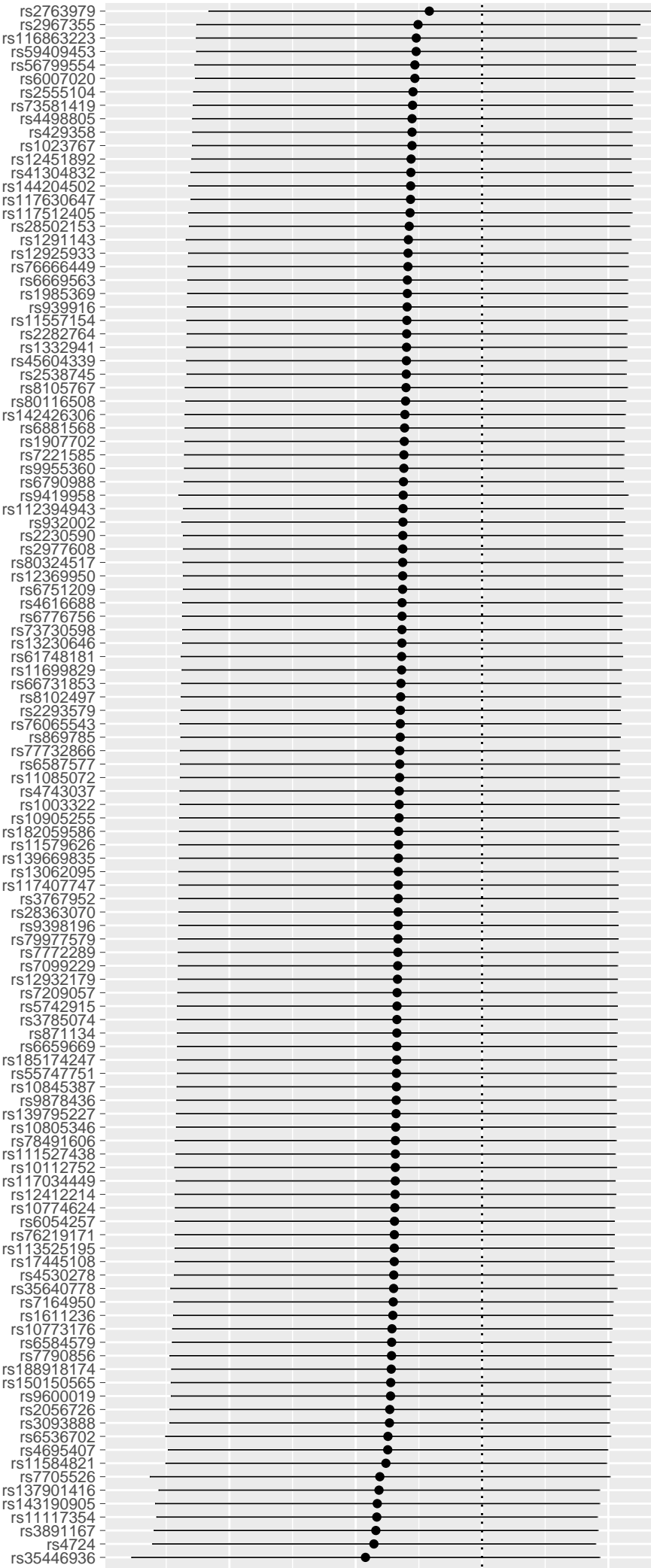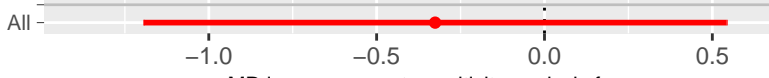

MR leave-one-out sensitivity analysis for 'telomere length || id:ieu-b-4879' on 'outcome'

Supplement: Supplementary file 1 [file nutrients-16-01417-s001.zip › Figures/Figure S3 TL to MGmet.pdf]

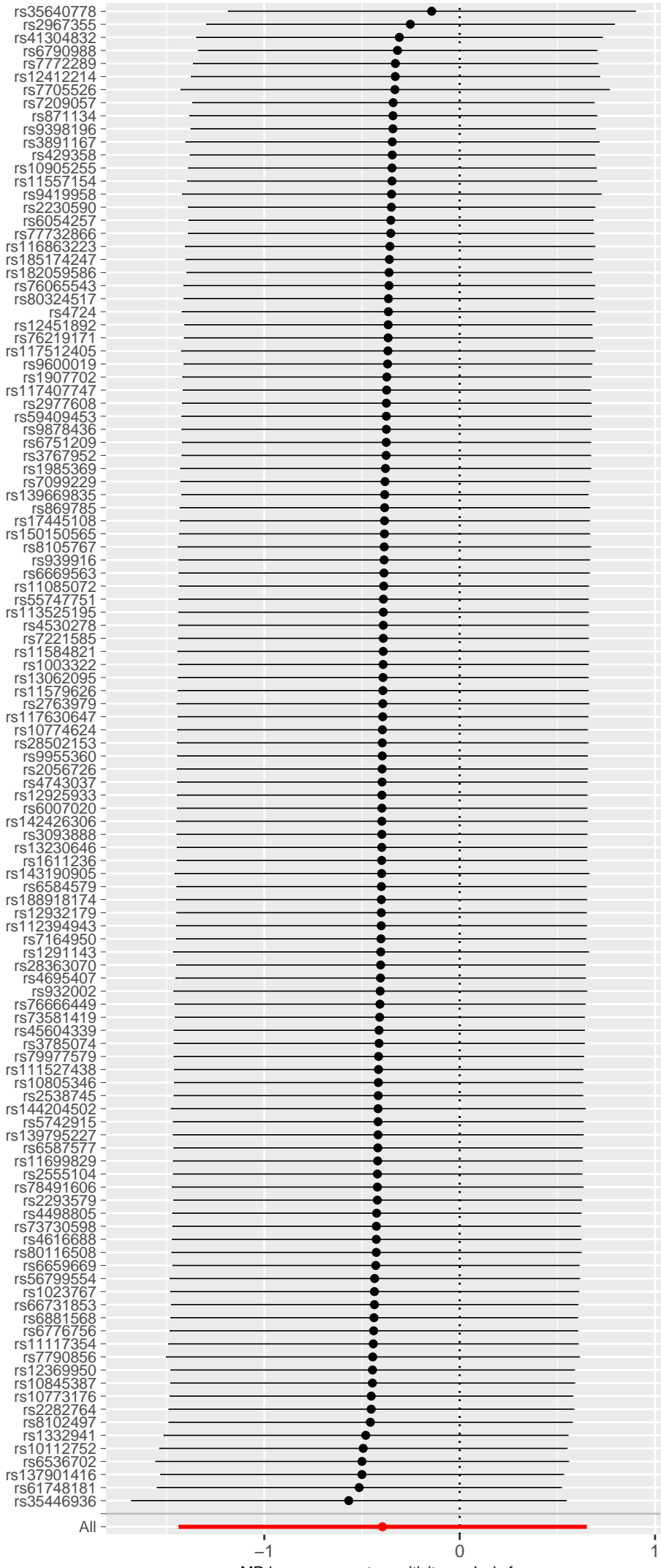

MR leave-one-out sensitivity analysis for 'telomere length || id:ieu-b-4879' on 'outcome'

Supplement: Supplementary file 1 [file nutrients-16-01417-s001.zip › Figures/Figure S4 TL to PHOS met.pdf]

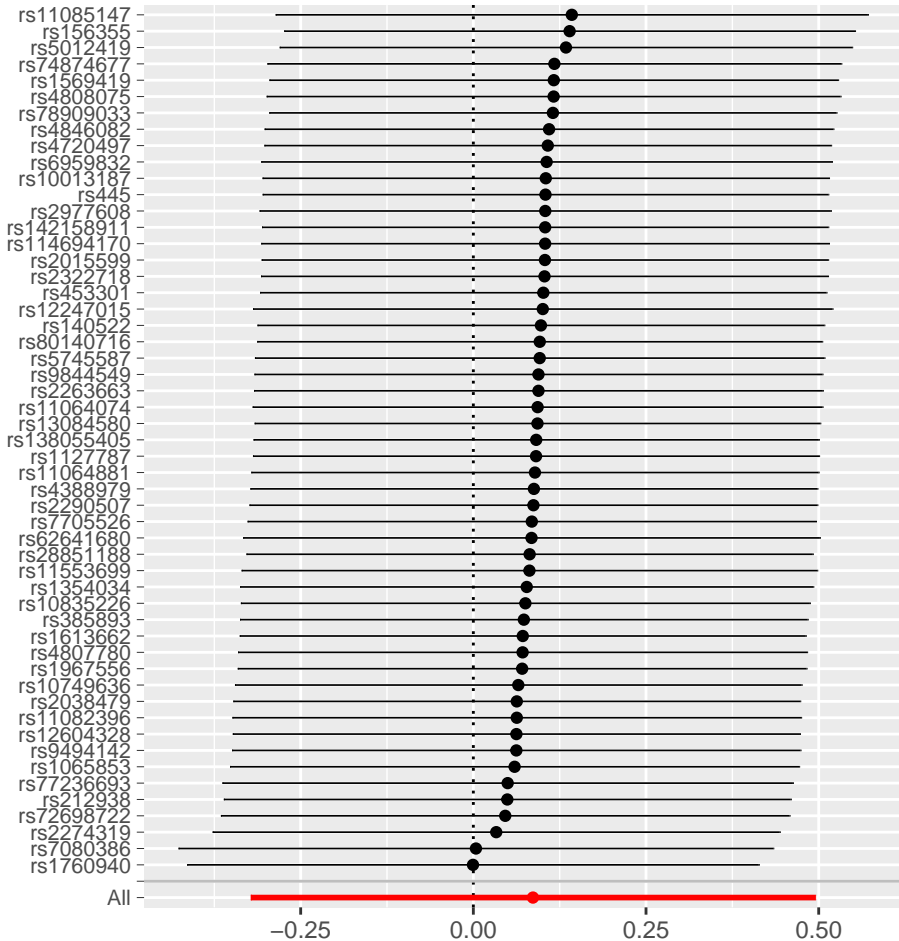

Supplement: Supplementary file 1 [file nutrients-16-01417-s001.zip › Figures/Figure S5 mtDNA_CN to minral met.pdf]

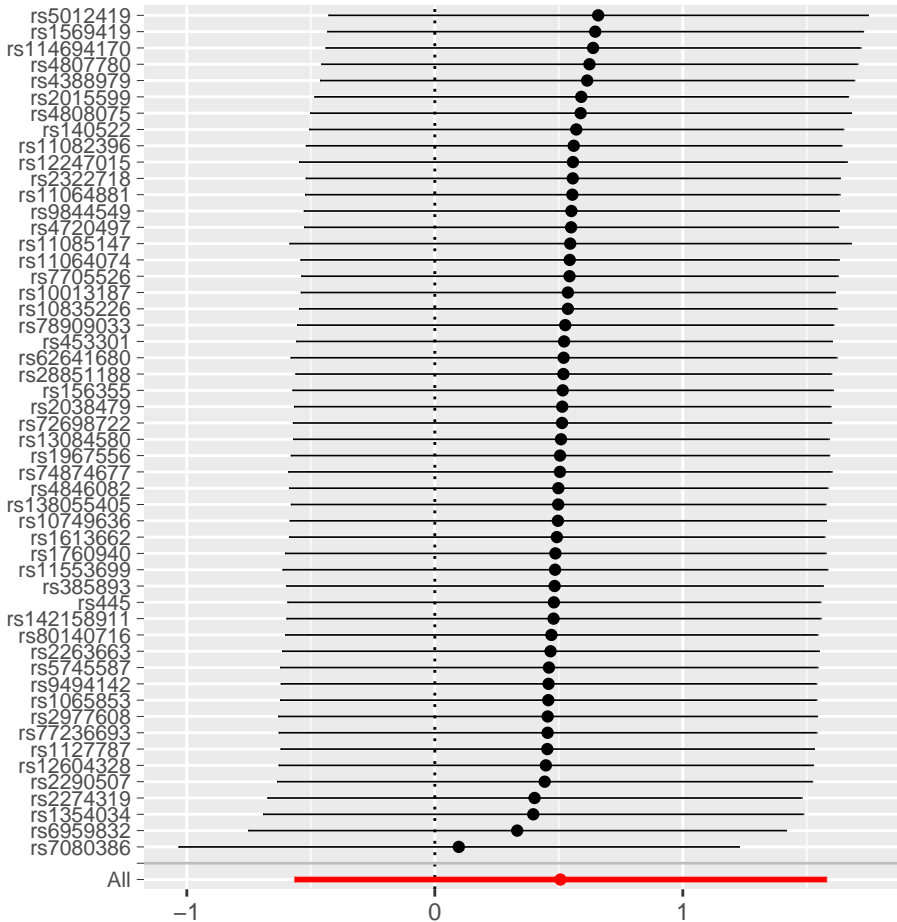

Supplement: Supplementary file 1 [file nutrients-16-01417-s001.zip › Figures/Figure S6 mtDNA_CN to IRON met.pdf]
